# Epidemics Scenarios in the “Romantic Network”

**DOI:** 10.1371/journal.pone.0049009

**Published:** 2012-11-27

**Authors:** Alexsandro M. Carvalho, Sebastián Gonçalves

**Affiliations:** 1 Instituto de Física, Universidade Federal do Rio Grande do Sul, Porto Alegre, Rio Grande do Sul, Brazil; 2 Centro de Ciências Exatas e Tecnológicas, Universidade de Caxias do Sul, Caxias do Sul, Rio Grande do Sul, Brazil; Universidad de Zarazoga, Spain

## Abstract

The networks of sexual contacts together with temporal interactions play key roles in the spread of sexually transmitted infections. Unfortunately, data for this kind of network is scarce. One of the few exceptions, the “Romantic network”, is a complete structure of a real sexual network in a high school. Based on many network measurements the authors of the work have concluded that it does not correspond to any other model network. Regarding the temporal structure, several studies indicate that relationship timing can have an effect on the diffusion throughout networks, as relationship order determines transmission routes. The aim is to check if the particular structure, static and dynamic, of the Romantic network is determinant for the propagation of an STI. We performed simulations in two scenarios: the static network where all contacts are available and the dynamic case where contacts evolve over time. In the static case, we compared the epidemic results in the Romantic network with some paradigmatic topologies. In the dynamic scenario, we considered the dynamics of formation of pairs in the Romantic network and we studied the propagation of the diseases. Our results suggest that although this real network cannot be labeled as a Watts-Strogatz network, it is, in regard to the propagation of an STI, very similar to a high disorder network. Additionally, we found that: the effect that any individual contacting an externally infected subject is to make the network closer to a fully connected one, the higher the contact degree of patient zero the faster the spread of the outbreaks, and the epidemic impact is proportional to the numbers of contacts per unit time. Finally, our simulations confirm that relationship timing severely reduced the final outbreak size, and also, show a clear correlation between the average degree and the outbreak size over time.

## Introduction

The progress in medical and biological sciences by the end of sec XIX eventually led to the identification of the organisms responsible for the diseases, making it possible for the systematization of mathematical descriptions for epidemics. While the first attempt to model mathematically epidemics was probably in 1760 by Daniel Bernoulli in his study of vaccination on the spread of smallpox [1], it was Hamer who introduced the idea of mass action [2] to model epidemics. Then Kermack and McKendrick used the idea which evolved into the present form of the susceptible-infective-removed (SIR) model [3], and its variants, so ubiquitous in the theoretical description of epidemics nowadays [4], [5]. As a mass action or mean field model, the SIR model (and its SIS, SIRS, etc, variants) assumes that all classes are superimposed in space. Even in the more elaborated case of space distributed classes (Fisher's equations) it is locally assumed the superposition of classes. However, in the last decade, it became clear that the transmission of a disease could be influenced by the contact network [6], [7]. Such a network depends on the structure of interactions among the individuals, which in turn may depend on the peculiar disease. Several articles have addressed the disease propagation problem of networks using paradigmatic examples such as small-world [8] or scale-free networks [9]. The high heterogeneity of some extreme topologies lead to important consequences such as the persistence of epidemics for any value of the spreading rate in an infinite scale-free network [10], [11]. The reason comes from the non epidemic threshold of the infinite-variance degree distribution of such infinite network, however human networks dubiously present such extreme behavior; for more details see Ref [12]. General results regarding the threshold, size and distribution of epidemic bursts were given for small-world networks in one and two dimensions [13].

While there have been recent efforts to incorporate human behavior into disease models (see ref [14] for a review), to implement disease-spreading agent based models, including attributes at real human populations sizes, is still a very difficult task [15]. Until that level of sophistication is a reality, the study of the spread of hypothetical diseases in real networks can be of great help. Until recently, the complete description of a real network —at least one that could be related to a specific disease— was something that did not seem feasible. Consequently, the only information at hand was statistical data such as the degree distribution of the network associated to the disease. That is the case of the Sweden study [16], in which a questionnaire addressing sexual behavior was completed by 4781 subjects. Unfortunately, that kind of questionnaire is not only subject to bias (for our purposes, the most relevant questions are about the number of partners in the last year and in a lifetime, the latter has a particularly strong bias [17]), but also does not allow us to build the real network behind the individuals, because the subjects represent a small sample of a larger population, the links of which are unknown. However, some years ago, the picture of a small, but real, sexual and romantic network became available. The “Romantic Network”, as called by the authors [18], is one of a few examples of a complete network, so simulation of epidemics with it can be very important, although its limited validity must be taken into consideration. There are other two networks more recently available and made from real data: the Likoma network [19] and the Internet sex network of Brazil [20]. However, from the figures of the first one it is not possible to code the network as we did with the romantic network. The second one was obtained indirectly from posts to a forum and restricted to sex commerce contacts. For a broad picture of the available human networks and how the data were obtained see Ref [21]. Moreover, information on the dynamic formation of links is available for the Romantic network [22], which makes this particular example even more interesting. If we take the static network and run a simulation of an epidemic, with some values of infectivity and infection time, at the end we can have for example 10% of the subjects infected. But if we take into account the dynamics of the formation of links, what will the final result be? In other words, how different will the final outcome be from the static scenario? There is no answer to that question without doing a simulation in the dynamic network and that is one of the principal aims of the present study.

Therefore, in the present work we implement and analyze computer simulations of the evolution of a hypothetical sexually transmitted infection (STI) in the Romantic network. From such simulations it is possible to evaluate the risk and impact of a potential epidemic in this and similar networks. The analysis is based on the specific structure of that network and it is compared to several well known model networks, which are potentially representative of human interactions. Once the network is established and known, one is faced with different schematic ways of conducting the disease spreading simulations. Consider an infected node with some other susceptible nodes connected to it. The different ways in which the contagion can go from this node to its neighbors can be schematically divided into three cases: at each time step (which can represent a day for example) it can be through only one link, through some of them simultaneously, or through all of them in the same period. Among the three possibilities and for matters of simplicity we chose the first one (one contact per time step) for all the simulations of the Results section, leaving one specific sub-section to discuss the other possible choices. In contrast to the usual approach of mass action models and static networks, several recent articles [23]–[27] have addressed the problem of disease propagation from the perspective of the dynamic formation of links. As was emphasized in those researches we believe that the timing present in all social networks is a key factor regarding the flow of information (disease, rumors, etc) that goes through them. Therefore one section of our study is devoted to this subject.

## Materials and Methods

### Empirical network

**Figure 1:**
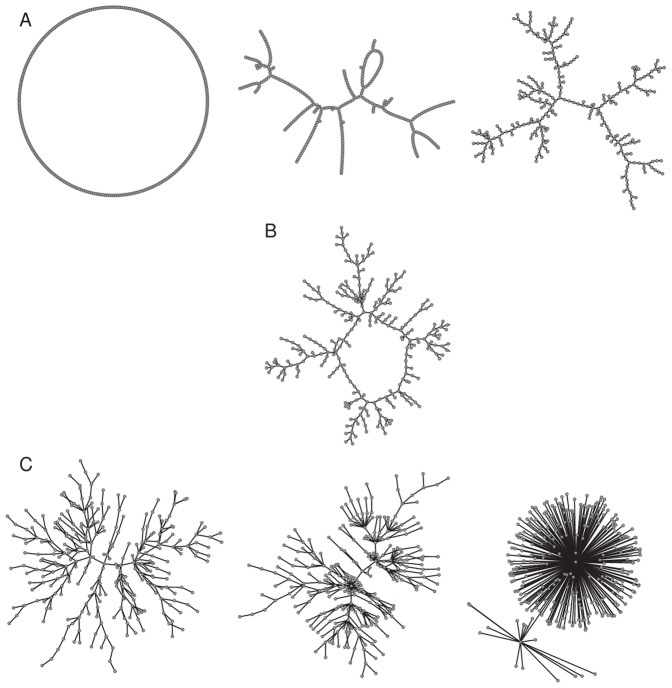
Networks realizations used in this work. (A) Watts-Strogatz: from left to right, 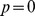 (ring), 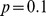, and 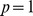 (random); (B) Romantic; (C) Krapvisky-Redner: from left to right, 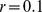, 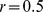, and 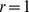. All of them have the same number of vertices, 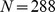, and the same average degree, 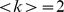. Figures generated with PAJEK [28].

**Figure 2:**
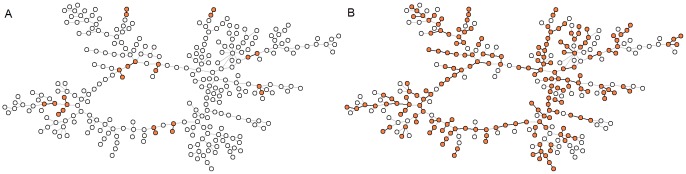
Snapshots of the network taken from frames at times (A) 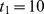 and (B) 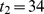. The hatched vertices correspond to the interacting individuals. Figures generated with PAJEK [28].

In 2004 the first picture of an almost complete sexual network of a social group was published [18]. This network, called by the authors “Romantic and Sexual Network” emerged from data taken from the National Longitudinal Study of Adolescent Health, a longitudinal study of students in grades 7–12 in the US. Among the 140 schools included in the study, 14 had saturated field-settings. In “saturated” cases **all** students of the school took the in-home interviews, which ask to nominate their partners, so complete sexual and/or romantic networks could therefore be constructed with these data. From those, one of the two largest ( 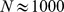) was used to depict the aforementioned network. The data was obtained after home interviews covering a period of 18 months, where students were asked to identify their sexual or romantic partners from a list of the student attending their school. “Jefferson High School” is the fake name of the only public high school in “Jefferson City”, a non-identified mid-sized mid-western town of the US, with a homogeneous (all-white, mostly working class students) and isolated community. Participating in the in-school survey were 90% of the school roster and a total of 873 completed the questionnaires. After data was collected a network of 573 nodes emerged, from which probably the most remarkable feature is a ring-like component involving 288 students. The 288 nodes component emerged as a result of superimposing all contacts among the students during the 18 month period, see Fig. 1B. However, taking into account the temporal sequence, James Moody published a dynamic version of this network in his site [22]. In order to see the evolution of an hypothetical infection both in the static and dynamic network, the animated figure cited above was broken into its 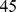 frames, each one corresponding to a period of 12 days, which for simplicity we assume evenly distributed in time. The nodes and links between them were identified and converted to input data for the simulation of the epidemic models. Each frame taken individually looks very different from the superposition of all of them (the giant static component). To give an idea of what it looks like, we show in Fig. 2 two frames at times 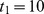 and 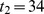, which represent times of low and high activity in the network, respectively. Therefore, it could not have been guessed before the picture was finished, nor by the researchers, neither by the participants. Besides, we manually mapped the graphical representation of the giant component (static and dynamic versions) and checked with Pajek [28] that our transcription gave the same graphical representation as the original. The “Romantic Network” has obvious limitations mainly due to its small size as compared to the networks of other important cases, such as the AIDS related sexual networks of South Africa for example. However, in contrast to those large networks examples, the Romantic network is an almost complete graph of a closed community on which we have extensive dynamic information, it is therefore an example worth studying.

### Artificial networks

For the artificial networks we use the Watts-Strogatz (W-S) [29] and Krapivsky-Redner (K-R) [30] which are good models for representing human relations. They cover many scenarios from ordered to disordered, and from homogeneous to heterogeneous, respectively, by means of one control parameter. They also allow for the easy control of the average degree. The W-S networks are implemented starting from a ring of 288 nodes linked up to first neighbors. We give the disorder parameter 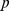 different values from 0 (the original ordered ring) to 1 (a random network), avoiding the break up of the component, see Fig. 1A. So all degrees of disorder are tested including the normally accepted small-world values (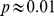). For the heterogeneous K-R networks we use the redirection procedure (see Fig. 1C): at every time step, a new node is added to the system and an earlier node 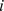 is selected uniformly, as a possible target for attachment. With probability 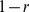 a directed edge from the new node to 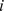 is created; with probability 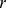 the edge is redirected to the ancestor node 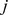 of node 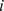. Starting from two connected nodes we let the network evolve until it reaches a 288 nodes component. This model leads to a power-law degree distribution —when it grows to large sizes— with degree exponent 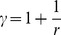, which can be tuned to any value equal or larger than 2. The real network is bipartite (145 males and 143 females), but for all the epidemic dynamics in the real and artificial networks we consider all nodes of the same kind, because we assume them to be non directional networks, without distinction between links. Apart from the size, the most relevant feature for the implementation of model networks which have to mimic the largest connected component of the Romantic network, is the average degree 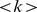. Its value is 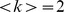, so all artificial networks were built with this same value.

### Simulation of epidemics

Epidemics in these networks are studied using computational simulations of the SIR model. For each specific network topology and realization —in which each vertex represents a person, with links representing the sexual relation—, one individual is randomly chosen to be the first infected, leaving the rest in the susceptible state. That is the initial state i.e., one node in the I state and the other 287 nodes in the S state. The simulation proceeds as follows: at each time step (which could represent one day or one week), each infected person chose a partner randomly from the list of subjects connected with him/her. If the chosen node is in infected or removed state, nothing happens, but if is in susceptible state it becomes infected with probability 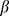. Such procedure mimics a sexual contact of two persons in which one of them has an STI. The simulation step is completed after all nodes have contacted one of its partners, so we assume that the sexual activity is homogeneous among the population and independent of the number of partners. We will discuss other options later. There are three random processes in the simulations which are implemented by the use of a pseudo-random number generator in the computer (we use Marsaglia random generator [31]): the selection of the first infected among the 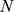 (288) nodes, the selection of one of the partners of each infected node at each time step, and the stochastic process of infection with probability 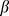. The first two are like throwing a dice, while the last one is implemented in a Monte Carlo (MC) procedure: by generating a random number uniformly in the [0,1) interval, and comparing it to 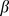, if the number is smaller (or equal) to 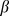, the infection occurs, otherwise not. Besides, a MC procedure is used previously to generate the W-S and K-R networks. Once infected, the subject remains so for a fixed period 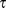 —being potentially contagious to its list of contacts as the dynamics proceed. After this period, the infected individual is removed (or becomes permanent immune). The process is repeated until no infected individual remains in the population. For a given situation, the disease parameters 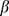 and 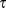 are fixed and have the same value for all subjects. The time step, as we mentioned before, can represent one day or one week or any other reasonable time period. If it is one day that means that every subject is having one intimate or sexual contact per day, which is probably an overestimation. If it is set at a week, it is probably too long. However the frequency related to sexual activity is absorbed in the 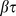 product, and a broad range of values will be tested in this presentation.

For the static scenario, the Romantic and artificial networks are characterized by the following quantities relevant to the SIR model: the final size of the epidemic, 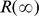, the maximum value of infected subjects or epidemic peak, 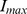, the time to arrive at that peak, 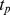, and the epidemic probability. All of these values were obtained by considering only the realizations with a final value of more than 5% of subjects infected. It is indeed the ratio between the number of those realizations over the total which we use to define the probability of an epidemic occurring. The number of different realizations for each kind of network (and for each value of 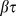) is two thousand, which corresponds to ten different graphs for each kind of network and two hundred different random sequences of the random number generator (i.e. different seed) used to simulate the infection.

Other possibilities in relation to the Romantic network are explored: external field, contact degree of the seed, and rules of interaction. By external field, 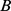, we mean the probability that a subject within the static empirical network interacts with an outsider, who is assumed to be infected. Therefore, the chosen subject, selected at random from the entire population, is infected with probability 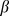. Obviously, 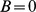 is equivalent to the normal dynamics without external excursions, while 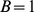 means that at each time step someone definitely encounters an infected outsider. 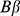 is then the likelihood of a spontaneous infection in the network. We studied the influence of the degree of the seed, i.e. how the number of connections the seed has and how that affects the spread of the disease. The rationale for this is that someone with many links should have more potential to infect than someone with only one link. We think this is a very important question to clarify before going on to epidemics in real situations. In order to test this, we defined the seed individual according to its degree, from the minimum value 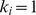 to the maximum 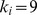, comparing them and the random selection of the seed. When 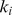 has its maximum value there is only one option for the seed, because only one subject in the static empirical network has this value. Then, as the degree of the seed goes down, i.e. exploring lower values of 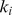, we have more options for the seed (there are more individuals with lower values of connectivity). In these cases we average outcomes over several initial choices of subjects. In all cases, we calculate these over many random number sequences, so even in the one possible initial case (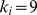) it represents the average of different possible “histories”, starting from this situation.

In real social dynamics the frequency of interactions is not uniformly distributed, i.e. one person could visit one or many of its contacts during a fixed period of time. In order to capture this feature in the epidemic models we face different possibilities when an infected subject interacts with their contacts; we call them rules of interaction. One: from all contacts, only one is chosen randomly at each time step. Fraction: a subset of the links is selected at random to contact at the same time step. All: all connected vertices are contacted at the same time step.

The dynamic network brings a new scenario. The inclusion of the dynamic formation of links modulates one of the basic properties of the network, the degree. In order to characterize the evolution of the dynamic network we measure (using the snapshots of the animated gif) the degree of each node over time and we use this data to obtain the dynamic degree distribution, 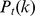. This distribution represents the probability that a randomly chosen node, at a given time 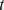, has degree 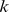. With this quantity, we follow the changes of the average degree over time, 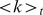. Since at different times, many sub-graph are disconnected from the giant component, another relevant measure is the size of the largest connected component, 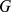. Lastly, we characterized the spread of the epidemic in this scenario. For this, we measure the fraction of infected nodes 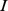. The dynamic study is done by separating the 45 frames that represent switching the links among the subjects on and off. Examples of these frames are the two snapshots depicted in Fig. 2 at times 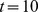 and 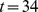. Each of these frames (i.e. the links between hacked nodes) are active for 12 days, after that the frame is replaced by the next one, and so on. The superposition of the 45 frames makes the giant component. As we can see in Fig. 2, the only possible path for the infection to propagate is through the active links, which are the ones between marked nodes. If the seed happens to be a node with no active links at that time, for the corresponding time window of 12 days the infection can not pass to anybody; in other words the propagation of the infection from the seed to one of its partners will only be possible when the link between them is active.

## Results

### Static Scenario

#### Romantic network vs artificial networks

**Figure 3:**
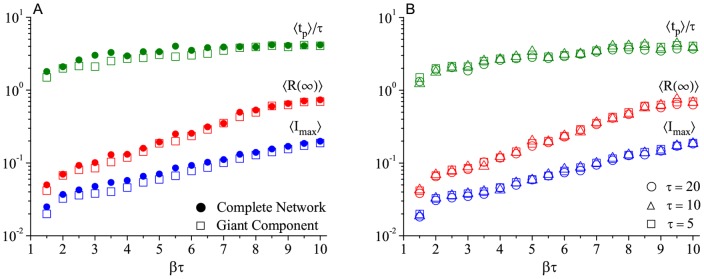
Behavior of the disease spread in the (static) Romantic network regarding. (A) Comparison between giant component and complete network (scaled by 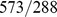, i.e. the *complete network/giant component* size relation) and (B) the 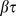 product. Each point of the curves is the result of an average over 200 independent runs (different sequences of the random number generator).

**Figure 4:**
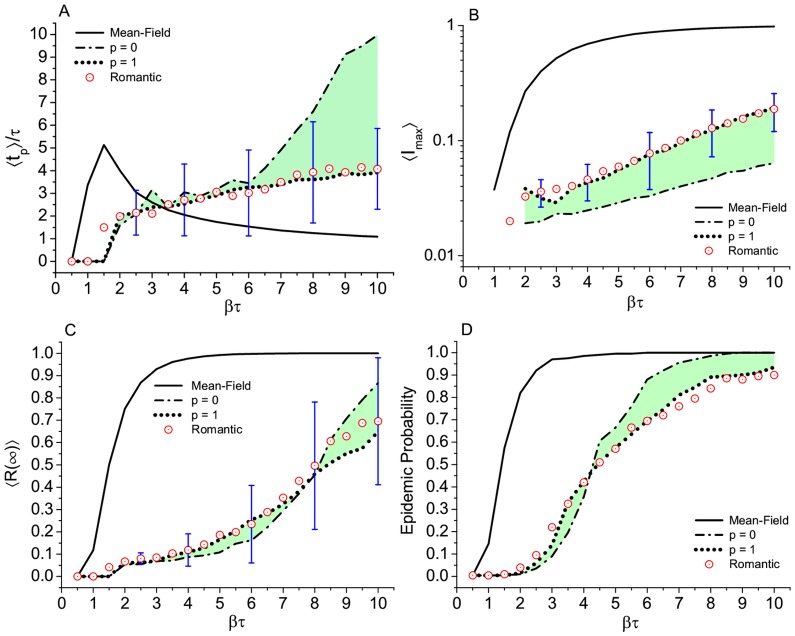
Epidemic simulation of the SIR model in different topologies. Watts-Strogatz, Romantic, and fully connected networks. Results for (A) time to the epidemic peak, (B) maximum number of infected subjects, (C) total number of removed or final prevalence, and (D) epidemic probability, defined as the fraction of realizations that end up with more than 5% of the population affected. Each point of the curves is the result of an average of over 200 independent runs on 10 different graphs (except for the Romantic network which is only one graph). Error bars, only displayed at some selected points, correspond to the standard deviation over the 200 runs in the Romantic network case. Green shadows help to visualize the variation of the outcome due to the progressive disorder in the W-S networks.

**Figure 5:**
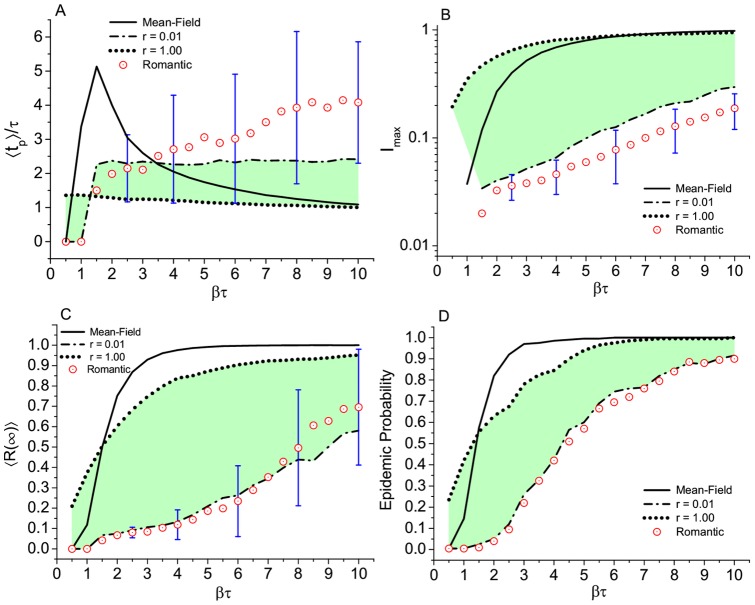
Epidemic in different topologies. Krapivsky-Redner, Romantic, and fully connected networks. Results for (A) time to the epidemic peak, (B) maximum number of infected subjects, (C) final prevalence, and (D) epidemic probability. Number of runs, average per point, and error bars as in Fig. 4. Green shadows help to visualize the variation of the outcome due to the progressive heterogeneity in the K-R networks.

Before presenting the results we want to make two important comments. In the graphic representation of the Romantic network originally presented in Bearman et al. paper [18], apart from the giant component of 288 nodes, there are many small components ranging from pairs, triplets up to ten nodes which give a total of 573 students. However any infection started in any one of those small components will be restricted to it. Consider the whole network of 573 elements will be equivalent to a renormalization of the giant component results by a 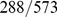 factor, which we have checked (see Fig. 3A). The second comment refers to Figure 3B, which clearly shows that different pairs of 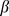 and 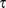 values, but with the same value of 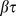, collapse to the same point. This is well known from the differential equation of the classical SIR model [5], however we want to show explicitly that it holds true for numerical simulations on static networks. So the statistical properties of disease outbreaks depend only on the product 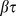 which, along with the degree distribution 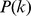, defines the theoretical value of the basic reproductive number 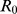. Therefore, our results will be shown in terms of 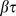 which, as expected, appears to be a good parameter to characterize all of them. From a general perspective, we observe that, except for the K-R networks with 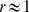 (the most heterogeneous networks), many topologies give similar epidemic results provided that 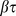 is below 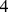. This can be concluded from the comparison between the Romantic and the W-S networks, as can be seen in Fig. 4, and the K-R for 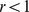, as shown in Fig. 5. Moreover, if we consider a small group of topologies, the W-S with large 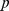 (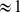) and the K-R with small 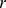 (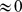), the behavior of the results, specially 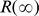 and the epidemic probability (Figs. 4,5 C,D) are very similar to the corresponding results from the Romantic network, up to values of 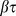 equal to 8. Considering individual realizations, the displayed error bars in Fig. 4A–C and Fig. 5A–C correspond to the Romantic network and give an idea of the observed dispersion associated to its small size. We observed that the mean field (all mixed) is the worst possible scenario from the epidemic point of view. Any other situations, i.e., any other network structure in which not everybody is connected even the K-R heterogeneous networks yield a less unfavorable picture. The only exception is the K-R network with 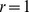 (which would give a scale-free network for an infinite system) but for values of 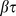 less than 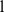. Comparing the outcome between the different networks and the Romantic network, it is possible to see that the latter does not fit into any of the models we have tried. Nevertheless, within the uncertainties of the fluctuation associated with the small (288 nodes) network considered here, an epidemic in a random network obtained with large 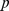 (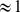) is statistically indistinguishable from an epidemic in the Romantic network. On the other hand, the topology that diverges more from the Romantic network (and from all the others) is the K-R network with 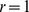. This particular value or r gives a power law distribution network with exponent 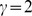 (scale-free) for large network sizes. In the present case, however, due to the small size of the networks, results in a star topology, very different from the ring-like type of the Romantic network.

#### External field

**Figure 6:**
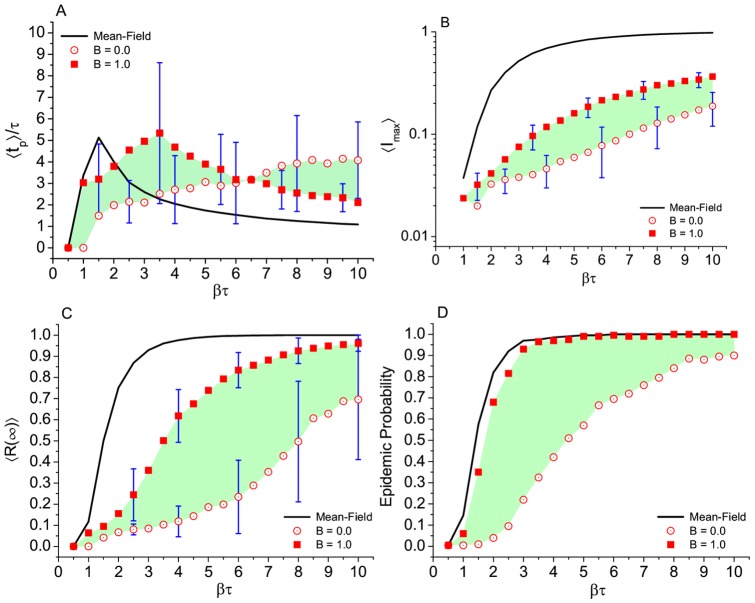
Effect of the “external field”, 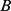, applied to the Romantic network. Results for (A) time to the epidemic peak, (B) maximum number of infected subjects, (C) final prevalence, and (D) epidemic probability. Comparison with standard mean field results in a network of the same size. Number of runs, average per point, and error bars as in Fig. 4. Green shadows help to visualize the variation of the outcome as increasing the 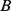 parameter.

The Romantic network was constructed taking into account all the sexual contacts of the surveyed school but excluding any possible contact outside the school. This section is devoted to exploring the effect of a possible contact of a member of the network with an infected subject from outside the network and the effect it can have in the epidemic dynamics inside the school. This effect is controlled by the parameter 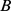 which represents the probability of the external excursion happening. As can be see in Fig. 6, the effect of the external field makes the outcome of the infection, measured by the total number of infected individuals as a function of 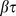, closer to what is expected from the standard SIR model. This suggests that the practical effect of the external field is to make the network similar to a fully connected network.

#### Degree of the seed

**Figure 7:**
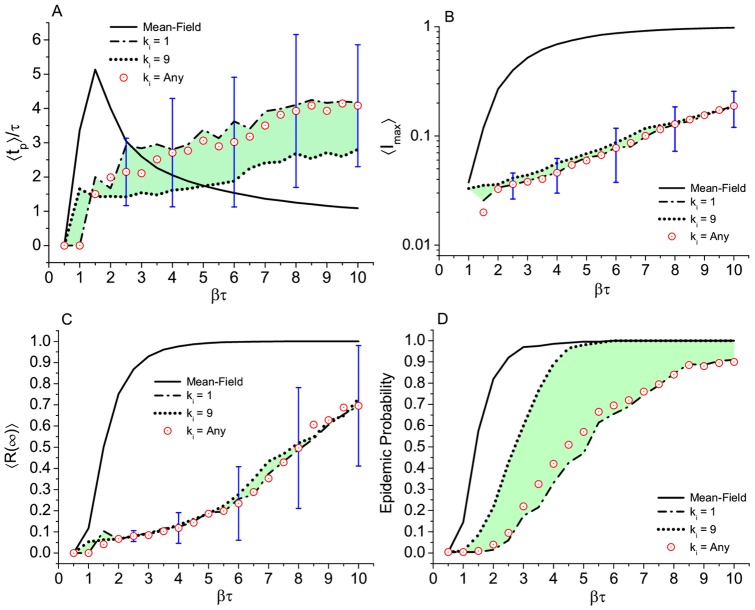
Effect of degree of the subject chosen to be the seed in the Romantic network. Results for (A) time to the epidemic peak, (B) maximum number of infected subjects, (C) final prevalence, and (D) epidemic probability. Comparison with standard mean field results in a network of the same size. Number of runs, average per point, and error bars as in Fig. 4. Green shadows help to visualize the variation of the outcome as response to the degree variation of the seed subject.

The results, presented in Fig. 7, are open to interpretation. Looking at Fig. 7B,C (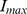 and 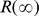 respectively) we are tempted to conclude that the influence of whom is the seed, if it is more o less connected, is irrelevant —which we have to admit is not obvious. However, the other two figures (Fig. 7A,D), representing the time to the infection peak and especially the last one, i.e., the epidemic probability, show a clear divergence from low (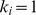) to high (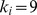) degree of the seed. At 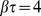 for example, we clearly see in Fig. 7C that, independent of who the seed was, the average number of subjects removed at the end is around 10%. Nevertheless, Fig. 7D remind us that in the low degree case that situation only happens in 30% of instances, in contrast to the 90% observed in the high degree case. Therefore, as expected, the degree of the seed is a key factor in the aftermath of disease propagation in the Romantic network.

#### Rules of interaction

**Figure 8:**
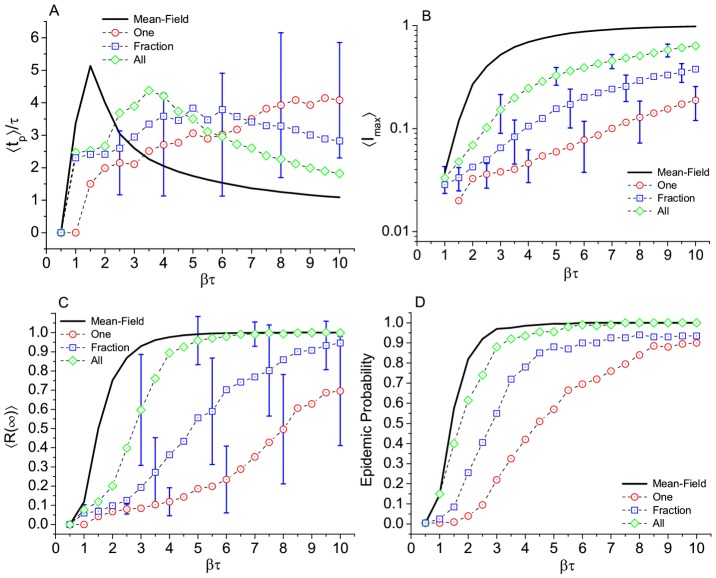
Effect of the different rules of interaction on. Results for (A) time to the epidemic peak, (B) maximum number of infected subjects, (C) final prevalence, and (D) epidemic probability. Comparison with standard mean field results in the same network. Number of runs, average per point, and error bars as in Fig. 4.

The three different measurements shown in Fig. 8 all point to the same general trend: from rule “one” to rule “all” we gradually approach the mean field all mixed case, which remains as the upper limit. Even the extreme “all” rule is below that limit, because subjects in the romantic network are not fully connected.

### Dynamic Scenario

#### Measurements

**Figure 9:**
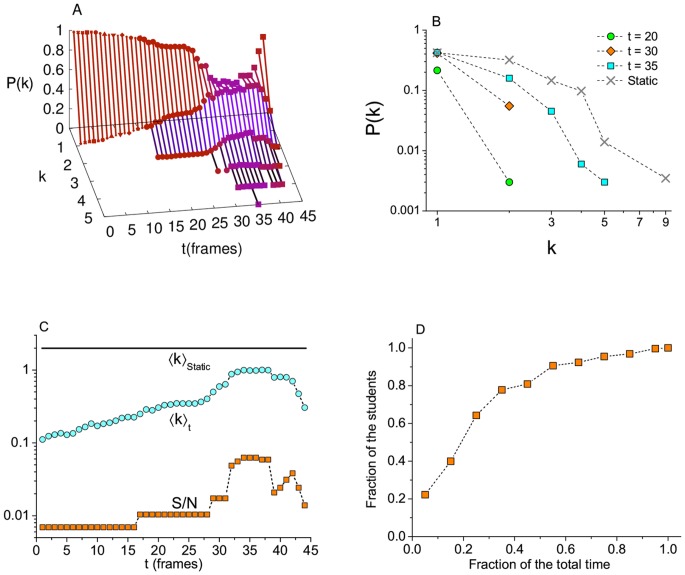
Characterization of the dynamic network. (A) Evolution of the degree distribution of the giant component of the Romantic network, when the frames are taken separately. (B) Degree distribution at three selected times (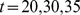) compared with the static degree distribution. (C) Average degree 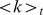 and largest connected component size 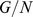 (normalized by 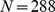) as a function of time. (D) Cumulative distribution of interaction times, as a fraction of the total time.

The Fig. 9A displays the degree distribution at different times. The inspection of that figure makes it clear that any measurement we can think of for networks will give very different values if applied to an individual frame, instead of the static network that results from the superposition of all the frames. That can be appreciated in Fig. 9B, where we compare the degree distribution at three different times around the most active frame, with the static network degree distribution. Over time, we have: for 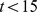 the interactions are only in pairs, with no connection between different pairs at the same time; the time window 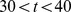 is where the activity increases to its maximum at 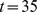; for 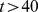 the degree distribution returns to low values. This is an extremely important point, because unlike some proposed models for the spread of epidemics in dynamic networks [25], [26], [32], this scenario can not be represented by an asymptotic degree distribution. The effect is greater over the average degree, 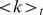, which has a peak value 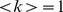 at 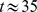, but most of the time it is much lower than the static value 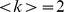 (Fig. 9C). Probably the most important effect of such measurements —in fact the reason for that difference— is that in all frames, many individuals have no contacts at all (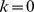). Following the behavior of random networks [33], we observed that the variation of the average degree directly influences the giant component size, 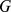. Another important measurement, related to the dynamic formation of links, is the fraction of time that each individual participates in the sexual activity, which is represented in Fig. 9D. That figure shows, for example, that while the majority of the students (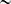
90%) participates less than 45% of the time, a small fraction of them (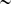
10%) are active for more than 55% of the total time.

#### Dynamic disease

**Figure 10:**
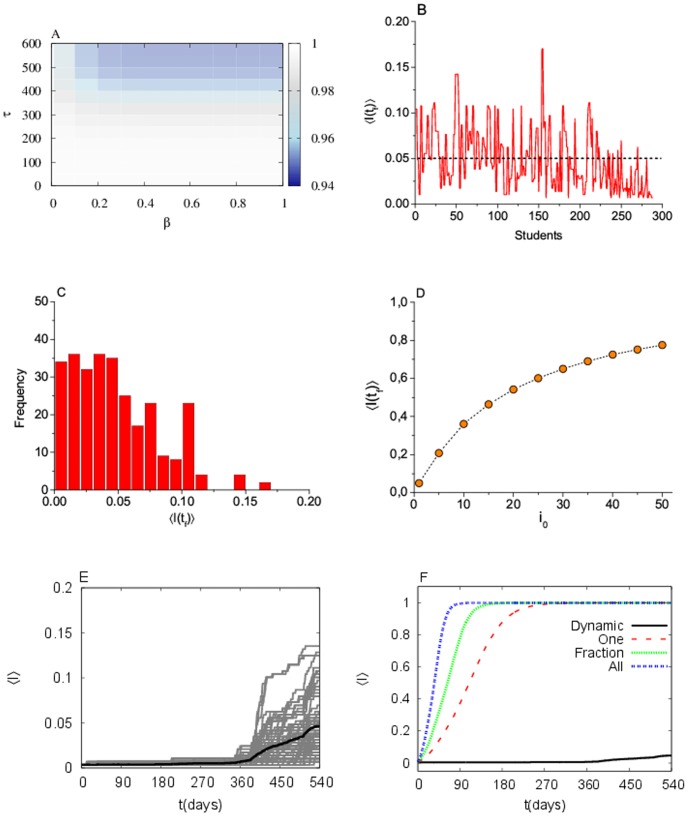
Epidemics simulations on the “Dynamic Romantic network”. (A) Final fraction of non-infected subjects 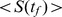 as a function of the infective time 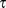 and the infection probability 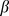 (white region means almost no infection); (B) distribution of infection size (fraction of total ever infected) depending on the seed subject; (C) histogram of the distribution (B); (D) average infection size (fraction of total ever infected) vs. the number of seeds; (E) fraction of infected subjects vs time; different runs with different seeds in gray, average in black; (F) average number of infected subjects vs time, comparison between dynamic network and static network with different dynamics rules. Each point of the curve is the result of an average over 200 independent runs. In figures B–F 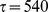 and 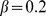 (notice however that figure A shows that for such value of 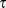 the dependence on 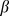 is weak above 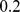).

The first result (see Fig. 10A,F) is remarkable: for the wide interval of 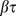 values explored, the final size of the epidemic is always much smaller than the corresponding static results. For the comparison to be possible we used the same unit of time (a day) for the time step in both cases; each of the 45 frames in the dynamic scenario lasts 12 days, which gives a total of 540 days. So, in the static scenario we use a total of 540 time steps. Practically, no epidemic will be observed in this scenario. In fact, only when the infection time 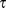 is close to the entire duration of the dynamics, we observe some effects in the susceptible population, although it is very weak indeed. Therefore, it is interesting to consider an extreme epidemic case, the SI epidemic dynamics, which means a lifetime infection time, so the relevant time is the duration of the dynamics. Some diseases, notoriously infection by HPV virus, have this kind of dynamics where 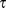 is equal to the lifetime. This is what is presented in Fig. 10 with parameters 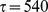 and 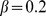, except in Fig. 10A where all possibles values of these two parameters are explored (up to the extreme value 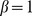, which means a certain infection every time an S-I contact is tried). Even with this extreme setup the total number of infected individuals at the end is under 15%. However, when simulations using every student as a seed are averaged, the number of infected subjects is not greater than 5% (see Fig. 10B and 10C). Fig. 10D shows the number of infected subjects at the end of the observation time as a function of the number of seeds, and we see that even with more than 50 over 288 seeds (17%) the final proportion of infected subjects is under 80%. All together these results strengthen the key role of the dynamic formation of pairs, which presents a novel scenario for modeling epidemics in networks. In the dynamic network, the total number of infected individuals is strongly affected by the initial condition, as can be seen in Fig. 10E. In this case, whoever is the seed is very important, not because of the personal activity, but due to the particular sequence in which the individuals connect to each other. It is the whole sequence which matters, not a particular initial subject. From the dynamics we see that the number of infected subjects grows substantially from 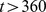. Figure 10F shows the comparison between the static and dynamic Romantic network within the SI disease. The difference is remarkable. The static network, for any of the transmission rules, is much more efficient for the disease propagation than the dynamic version.

## Discussion

In this study we addressed the problem of STI epidemics in a real network (the so called “Romantic or Jefferson School network”) in two ways. First, to compare epidemics in this network with epidemics in model networks to see whether the latter (or which one of them) represent a real network. Particularly, if the disease propagation is sensitive enough to details in the topology. The second aspect, the effect of the dynamic formation of links and how this can affect the epidemic dynamics, is far more relevant, especially because of the conclusions we have drawn. Regarding the static network and the comparison between the Romantic network and the model networks, the maximum incidence and the final size or prevalence of the epidemic are sensitive to the topology for relatively large values of 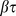 (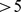). For smaller values of 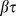 (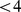), the different topologies —except the high heterogeneous ones— are hard to distinguish in terms of epidemic outcome. Even more if we consider the wide dispersion of the overall results due to the small size of the networks (bars in Figs. 4,5). Homogeneous networks, like the Watts-Strogatz with 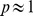 and the Krapvisky-Redner with 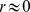 are statistically similar to the Romantic network in terms of epidemic outcome. This is valid when 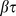 is not very large and it is due to the peculiar average value of the degree (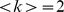) which makes all the structures ring or tree like, as can be seen by direct eye inspection of Fig. 1 (A-right, B, and C-left). The main difference between these artificial networks and the real network is the ring structure. However, there is only one relatively large ring. Among all artificial static networks tested in this study, we can conclude that the Watt-Strogatz network with 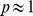 (random network with 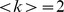) is indistinguishable from the Romantic network in terms of disease spread, for values of 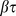 up to 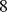. A marginal conclusion is that any network structure, in which not everybody is connected, including the K-R heterogeneous networks, gives a less severe scenario than the mean field all mixed result. The only exception is the K-R network with 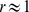 (and for 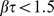) because it approaches a scale-free network. Considering the external field, the degree of the seed, and rules of interactions, our results suggest that the epidemic dynamics is affected by all of these variants. Regarding the external field, an individual contacting an infected subject from outside the network brings the results closer to that of the fully connected network. Concerning the degree of the seed (or the sexual activity of the starter of the infection) its importance is confirmed in the Romantic network. At 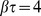 for example, the chances of a burst involving more that 5% of the subjects triples when going from the least to the most connected as the seed. The higher the degree of the seed the shorter the time to reach the peak of infection. Our results on frequency of contact (rules of interactions) confirm that the spread of an epidemic not only depends on the number of contacts available, but also on the frequency of contact with them [34], and as the frequency increases the results approach the mean field results.

In the dynamic network situation, that is considering the sequence of connections and disconnection between the subjects, we arrive at very interesting conclusions. We have shown with simulations (the only possible tool in this case) that in the dynamic network we always observe less severe epidemics. Not only because are there periods in which we see only small isolated components (at those times the infection can not jump from one component to the others), but the time windows in which they are connected (and not all at the same time) are relatively short. In other words, relationship timing directly influences the course of the epidemics [24]. Clearly, the propagation of a disease that depends on the links, which are created for definite time intervals, will be strongly affected by such dynamical processes. We have seen that the average degree in the dynamic case has a peak of value 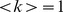, but usually it is much lower than the static value 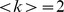. Besides, in the dynamic case the distribution of interaction times, absent in the static case, is crucial for disease propagation. By testing a hypothetical extreme epidemic situation, the Susceptible-Infective model with maximum infection probability, we obtained a final number of infected subjects of around 15% in the worst case (starting the infection with the most connected subject). However, the average number of infected subjects, from all the possible choices of the seed student is not bigger than 5%. Put together, these results show the key role of the dynamic formation of pairs, which brings a different scenario from previous results in static networks. Remarkably, in the dynamic network the total number of infected individuals is strongly affected by the initial condition. In this case, whoever the seed is, is important, not due to personal activity, but because of the particular sequence in which the individuals connect among themselves. It is the whole sequence that matters, not the particular subject. Moreover, comparing Fig. 9C and Fig. 10E we see a clear correlation between them. Thus, the dynamic of the largest component is related to the evolution of the fraction of infected subjects. Like a random network, the giant component follows a close relation with the average degree, as can be seen comparing 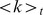 and 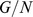 in Fig. 9C. This leads us to conclude that the fraction of infected people is related to the dynamics of the average degree, which can be important for public health. Additionally, we observed that the static network, in any of the transmission rules, is much more efficient for the disease propagation than the dynamic version. The effect of the dynamic formation (and break) of links is quite clear: A disease that depends on the structure of the links which are not available all the time, but in very specific time windows, will be influenced by them; but in a “desirable way”, because the worst scenario is always the static one, when all links are available all the time.

Although our results are of a restricted applicability, because of the small size of the network, we emphasize that the Romantic network is one of the rare examples of real and complete sexual network of a whole (closed) community. So even though we can not make a direct extrapolation of all the present results to a larger sexual network, like the AIDS related ones of South Africa for example, we think that our conclusions, regarding the similarity with random networks and the key role of the dynamics modulating the epidemic outcome, are important results with probably general validity.
